# Ubiquitin Proteasome System in Stress and Disease

**DOI:** 10.1155/2012/454796

**Published:** 2012-11-26

**Authors:** Dmitry Karpov, Michael H. Glickman, Shoshana Bar-Nun, Philip Coffino

**Affiliations:** ^1^Laboratory of Chromatin Structure and Functions, Engelhardt Institute of Molecular Biology RAS, Moscow 119991, Russia; ^2^Department of Biology, Technion – Israel Institute of Technology, 32000 Haifa, Israel; ^3^Department of Biochemistry and Molecular Biology, The George S. Wise Faculty of Life Sciences, Tel Aviv University, 69978 Tel Aviv, Israel; ^4^Department of Microbiology & Immunology, University of California, San Francisco, CA 94143-0414, USA

The proteasome is an ATP-dependent multisubunit self-compartmentalised protease complex. It is the major regulator of intracellular proteins turnover. The majority of proteins degraded by proteasomes are modified by covalent attachment of polyubiquitin chains. Substrates include regulatory proteins, transcription factors, components of signal transduction pathways, and proteins that have aberrant structures. The ubiquitin-proteasome system (UPS) thus participates in regulating diverse and numerous cellular processes. Identifying novel proteasome substrates and understanding the mechanisms of proteasome-dependent protein degradation require careful planning and implementation of methods for measuring the rate of protein degradation. Beatriz Alvarez-Castelao et al. discuss state-of-the-art methodologies for investigation of protein degradation. They focus on sources of potential experimental pitfalls and suggest ways to avoid them. 

Apart from ubiquitin, there is a large family of ubiquitin-like protein modifiers that are also involved in multiple forms of proteolysis-dependent and proteolysis-independent regulation. The SUMO (small ubiquitin-like modifiers) family is a well-studied example. Investigation of mechanisms of SUMO-dependent processes requires identification of the sites of SUMOylation and/or SUMO-interacting proteins and sequences. Elisa Da Silva-Ferrada et al. present a survey of the current methodologies used to study SUMO-regulated functions and identification of *cis* and *trans* sequences controlling SUMOylation. Based on critical consideration of previously described methods, they provide guidelines for selection of the appropriate method depending on the research goal.

If polyubiquitination mainly serves for proteasome-dependent protein degradation, protein monoubiquitination may be read as a signal of proteasome-independent regulation of protein activity. A well-known example is the internalization and endosome sorting of membrane receptors. Michel Becuwe et al. describe mechanisms of action of arrestins, a highly conserved protein family and one of the key regulators of G-protein coupled receptors (GPCR) signaling. Arrestins may regulate GPCR activity by serving as ubiquitin-ligase adaptors. It should be noted that Robert J. Lefkowitz and Brian K. Kobilka were awarded the 2012 Nobel Prize in Chemistry for their pioneering studies of GPCR. Interestingly, arrestins themselves are regulated by ubiquitination/deubiquitination. A recently found family of arrestin-like proteins is involved in regulating the activity of membrane transporters and other membrane-bound proteins. For example, they can participate in proteolytic activation of membrane-bound precursors of transcription factors.

The UPS system has its own quality control of substrate ubiquitination. This function is performed by a class of deubiquitinating enzymes. Misregulation of deubiquitination may be a significant factor in cancer progression. Jennifer Hurst-Kennedy et al. have reviewed the current status of our knowledge on the role of an unusual deubiquitinating enzyme, ubiquitin C-terminal hydrolase L1 (UCH-L1), in tumorigenesis. Normally, UCH-L1 is an abundant neuronal protein but its expression level is also increased in malignancies. The authors suggest that UCH-L1 may serve as an early biomarker of malignancy as well as a potential therapeutic target.

UPS dysfunction may contribute to human disorders, and among them are severe neurodegenerative diseases. Sabine Schipper-Krom et al. describe the UPS involvement in progression of Huntington's disease. The N-terminal fragments of huntingtin (N-htt), which contain polyQ repeats, are prone to form aggregates and serve as the sources for toxic polyQ oligopeptides. This review focuses on two controversial issues, the impairment of UPS sequestered into htt aggregates, and the degradation by the proteasome of polyQ-containing proteins. The authors conclude that N-htt does not directly affect proteasome activity and proteasomes are not irreversibly sequestered into htt aggregates. Possible therapeutic means to augment proteasome activity against polyQ-containing proteins are suggested.

These review papers represent an exciting, insightful observation into the state-of-the-art, as well as emerging future topics. We hope that this special issue would attract a large attention of the peers and inspires them to conduct fruitful experiments. We would like to express our appreciation to all the authors and reviewers for their great support that made this special issue possible.

*Dmitry Karpov* *Michael H. Glickman* *Shoshana Bar-Nun* *Philip Coffino*

